# An extract from the Atlantic brown algae *Saccorhiza polyschides* counteracts diet-induced obesity in mice via a gut related multi-factorial mechanisms

**DOI:** 10.18632/oncotarget.18113

**Published:** 2017-05-23

**Authors:** Patricia Huebbe, Sibylle Nikolai, Anke Schloesser, Diran Herebian, Graeme Campbell, Claus-Christian Glüer, Annette Zeyner, Tobias Demetrowitsch, Karin Schwarz, Cornelia C. Metges, Thomas Roeder, Gerhard Schultheiss, Ignacio R. Ipharraguerre, Gerald Rimbach

**Affiliations:** ^1^ Institute of Human Nutrition and Food Science, University of Kiel, Kiel, Germany; ^2^ Department of General Pediatrics, Neonatology and Pediatric Cardiology, University Children’s Hospital, Heinrich-Heine-University Düsseldorf, Düsseldorf, Germany; ^3^ Section Biomedical Imaging, Department of Radiology and Neuroradiology, University of Kiel, Kiel, Germany; ^4^ Institute of Agricultural and Nutritional Sciences, Group Animal Nutrition, Martin Luther University Halle-Wittenberg, Halle, Germany; ^5^ Institute of Nutritional Physiology ‘Oskar Kellner’, Leibniz Institute for Farm Animal Biology, Dummerstorf, Germany; ^6^ Institute of Zoology, University of Kiel, Kiel, Germany; ^7^ Animal Welfare, University of Kiel, Kiel, Germany; * These authors share the first authorship

**Keywords:** circulating bile acids, bile salt hydrolase activity, intestinal gluconeogenesis, life span, mTOR activation, Gerotarget

## Abstract

In this study we addressed the questions whether an Atlantic brown algae extract (BAE) affects diet induced obesity in mice and which would be the primary targets and underlying key mechanisms.

Male C57 BL/6 mice were fed a hypercaloric diet, referred to as high fat diet (HFD), supplemented with a freeze-dried aqueous BAE from *Saccorhiza polyschides* (5 %) for 8 months. Compared to the control group, dietary BAE supplementation significantly attenuated increase in body weight and fat mass. We observed apparent metabolic improvement including normalization of blood glucose, reduced plasma leptin, reduced fecal bile salt hydrolase activity with lower microbial production of toxic bile acid metabolites in the gut and increased systemic bile acid circulation in BAE-fed mice counteracting adverse effects of long term HFD feeding. Survival of mice receiving dietary BAE supplementation appeared slightly enhanced; however, median and maximal life spans as well as hepatic mTOR activation were not significantly different between BAE and control mice.

We suggest that the beneficial metabolic effects of our BAE are at least partly mediated by alterations in gut microbiota associated with fermentation of indigestible polysaccharides that are major components of brown algae such as alginates and fucoidans. We moreover propose a multi-factorial mechanism that involves profound alterations in bile acid homeostasis, changes in intestinal and systemic glucose metabolism likely including increased intestinal gluconeogenesis, increased activity of the intestinally derived hormone GLP-1 contributing to promote systemic insulin sensitivity, and inhibition of α-amylase activity, which expectably limits dietary carbohydrate digestion and glucose release.

## INTRODUCTION

The World Health Organization states that in the past 30 years the worldwide obesity rates have nearly doubled reaching a critical number of 1.4 billion people in 2008 emphasizing a serious health problem. The imbalance between energy expenditure and intake caused by the sedentary life style and the energy dense diet rich in saturated fat and sugar is blamed for the pandemic. In search of prevention strategies, rodent models may provide valuable information on molecular targets and metabolic consequences of diet induced obesity. Commonly used mouse strains such as the C57Bl/6 mice exhibit substantial changes in lipid and glucose metabolism on a hypercaloric high fat diet (HFD). Compared to a standard low fat diet, these changes include massive weight gain and fat deposition in various tissues and organs, reduced insulin sensitivity as well as adipose tissue and liver inflammation [1, 2]. Only recently it has been shown that HFD feeding also significantly alters bile acid homeostasis by reducing the level of circulating bile acids [3] while increasing the concentration of bile acids in the intestinal lumen [4], which are understood as mechanistic components of the HFD induced pathology. The *ad libitum* intake of a HFD in mice and primates is associated with the premature occurrence of age related cellular senescence and in general with an accelerated ageing phenotype [5-7].

Populations with a long tradition of dietary use of seaweed such as in Japan and Korea possess lower obesity rates compared to people from the United States and Europe (http://www.worldobesity.org). Similarly, in mice the supplementation with brown algae extracts leads to a reduction in body weight, adipose tissue and liver triglycerides [8-11]. Algae extracts have been reported to normalize plasma lipids and glucose metabolism, and induce fatty acid oxidation in liver and skeletal muscle of HFD fed mice and rats [12-15]. Most of these studies used ethanolic extracts of Asian brown algae species from Korea, Japan and Malaysia that were administered via oral gavage over a relatively short period of time. Others focused on individual compounds or compound classes, such as fucoxanthin or phlorotannins, isolated from seaweed investigating their potential anti-obesogenic, lipid lowering and anti-diabetic properties in HFD fed mice [16-19].

Seaweed is generally low in fat but high in dietary fiber and mineral content [20] with numerous additional functional ingredients. Compared to other seaweeds, brown algae species, e.g. *Saccorhiza polyschides,* possess lower protein and higher polysaccharides concentrations [20]. The main polysaccharides in brown algae are alginates and fucoidans that are predominantly resistant to mammalian digestion enzymes but to a certain extent may be fermented by colon bacteria. Alginates are composed of 1,4-glycosidically linked uronic acid units (mannurate and gulurunate residues) that solubilized in water form viscous gels [21]. Fucoidans on the other hand, are sulfated fucose containing heteropolymers that, in relation to their chemical structure, have been shown to exhibit diverse bioactive effects [22, 23]. Fucoidans, alginates and also phenolic compounds from brown algae have been shown to decrease carbohydrate and protein digestibility by inhibition of digestive enzymes, e.g. amylase, glucosidase, pepsin and pancreatin, and by formation of insoluble resistant complexes [24]. There is great diversity among the phenolic compounds present in seaweed with polymers of phloroglucinol such as phlorotannin and dieckol attracting most scientific attention. Furthermore, carotenoids and sterols such as fucoxanthin and fucosterol as well as other lipids are highly abundant [25]. Since more than two decades the potential bioactivity of marine algae has been studied and lately anti-obesogenic and anti-diabetic effects have become of major interest. Among the molecular targets of algae extracts and isolated compounds are genes encoding proteins centrally involved in cholesterol and lipid synthesis, fatty acid oxidation and energy metabolism [8, 14, 16, 17]. Despite the knowledge of their beneficial metabolic effects, it is unclear whether the described tissues and pathways are primary targets of algal compounds or whether the metabolic improvement may be mediated indirectly by other mechanisms.

In the present study C57Bl/6 mice were fed a high fat and sugar diet supplemented with an aqueous extract of the Atlantic brown algae *Saccorhiza polyschides* over a period of 8 months. Beside phenotypic parameters including weight gain, body composition, energy intake and excretion we determined circulating, urinary, fecal and hepatic bile acids, the regulation of glucose metabolism and intestinal gene expression. Furthermore, the activation of age-related signaling pathways and the survival of 18 months old mice supplemented with the brown algae extract were studied.

## RESULTS

### Diet induced obesity and related pathologies are attenuated in BAE supplemented mice

**Figure 1:**
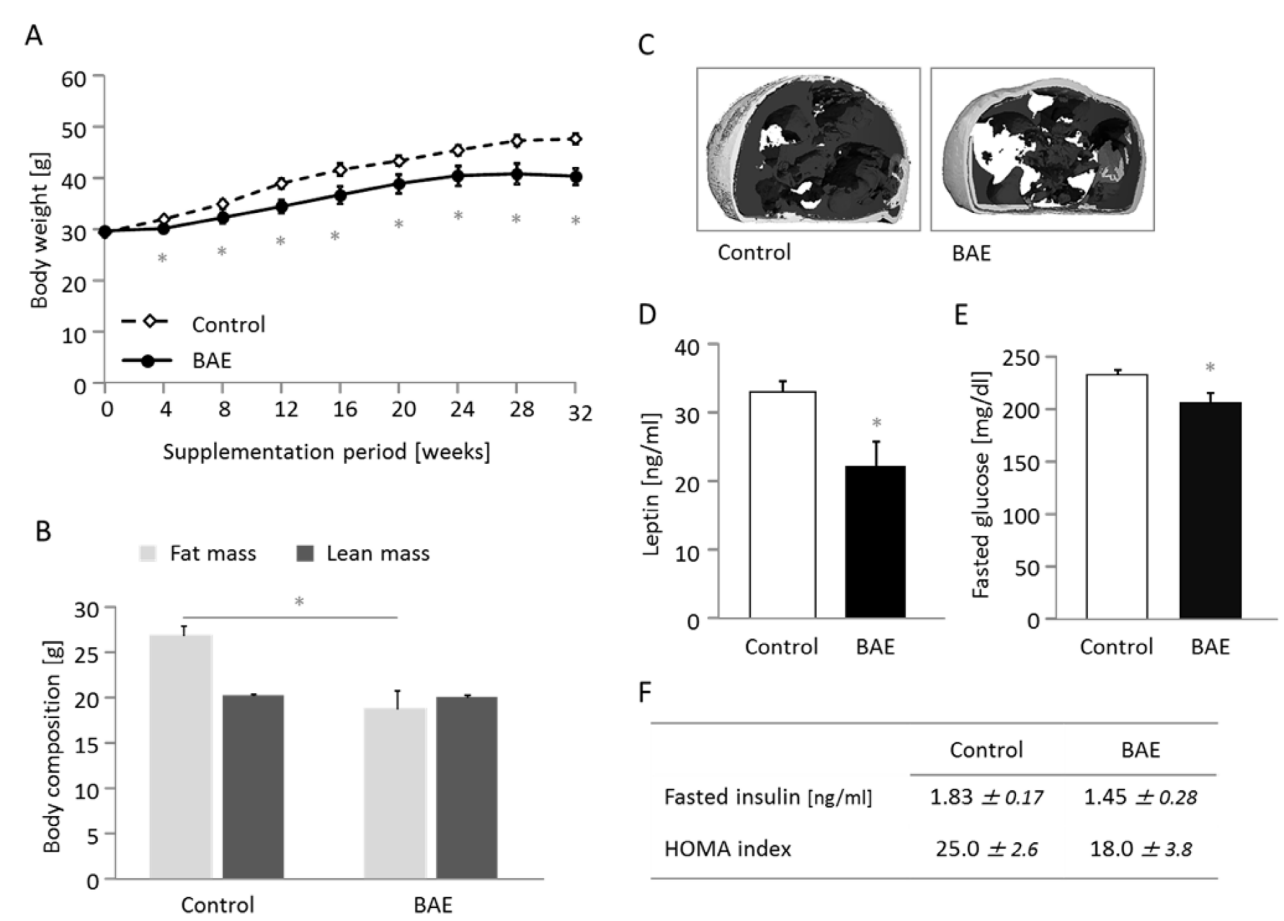
The brown algae extract (BAE) reduces body weight gain **A.** and fat mass **B.** and improves the metabolic phenotype **C.**-**F.** in mice fed a high fat diet. Abdominal adiposity (C) is illustrated in two images from the micro-computed tomography of mice representative for the differences between groups. Subcutaneous fat is illustrated in light grey and visceral fat as dark grey area. Fasted plasma leptin (D) and glucose (E) concentration were significantly lower, while insulin and the HOMA index showed a downward trend in BAE mice. Data are means ± SEM (*n* = 5 - 8). Statistically significant differences (*p* < 0.05) are indicated as *.

Compared to the control group, supplementation of the high-fat diet with BAE significantly attenuated body weight gain (Figure 1A) and accretion of fat mass (Figure 1B). The BAE fed mice appeared leaner and exhibited lower levels of abdominal adiposity and, in particular, of visceral fat deposition (Figure 1C). Food intake was higher in the BAE group, but because the energy density of the BAE supplemented diet was lower, the daily consumption of energy was similar between groups (Table 1). Excretion of fecal dry matter, water and energy was 20, 36 and 17 % higher, respectively, in the BAE supplemented group, although differences between treatments were not significant. Relative fecal energy density and apparent food digestibility did not differ between groups (Table 1). Consumption of drinking water was significantly increased in BAE vs. control mice (29.1 ± 2.2 vs. 34.7 ± 2.7 ml/week) and output of urine followed the trend (7.2 ± 1.4 vs. 11.0 ± 1.2 ml/week, *p* = 0.068). This was most likely due to the increased sodium content of the BAE compared to the control diet (7.3 vs. 4.8 g/kg).

**Table 1:** Food intake and fecal excretion is increased in high fat diet fed mice supplemented with a brown algae extract (BAE)

	Control	BAE
Food intake [g/d]	3.0 *± 0.03*	3.1 ± 0.03*
Energy intake [kJ/d]	62.9 *± 0.6*	61.6 *± 0.6*
Feces dry weight [g/wk]	1.7 *± 0.07*	2.0 *± 0.15*
Fecal water content [g/wk]	0.28 *± 0.02*	0.38 *± 0.07*
Fecal energy [J/wk]	29.7 *± 1.4*	34.7 *± 2.7*
Relative fecal energy [J/g]	17.0 *± 0.2*	17.0 *± 0.1*
Apparent food digestibility [%]	91.0 *± 0.3*	90.8 *± 0.4*

Mice were placed in metabolic cages for seven days. Daily food intake was recorded and individual fecal droppings were collected and pooled for later analysis. Data are means *± SEM* (*n* = 7 - 8). Statistically significant (*p* < 0.05) differences are indicated as *.

In addition, BAE mice showed significant metabolic improvement with lower leptin (Figure 1D) and higher adiponectin plasma levels (Supplementary Table 1). Likewise plasma glucose levels were significantly lower in BAE fed mice (Figure 1E), while insulin and HOMA index followed the same numerical trend (Figure 1F). Plasma total cholesterol, triglycerides and activity of AST and ALT (transaminases used as indicators of liver function) were not significantly different between groups (Supplementary Table 3).

### BAE supplementation alters microbial metabolism of bile salts and elevates the concentration of bile acids in systemic circulation

**Figure 2:**
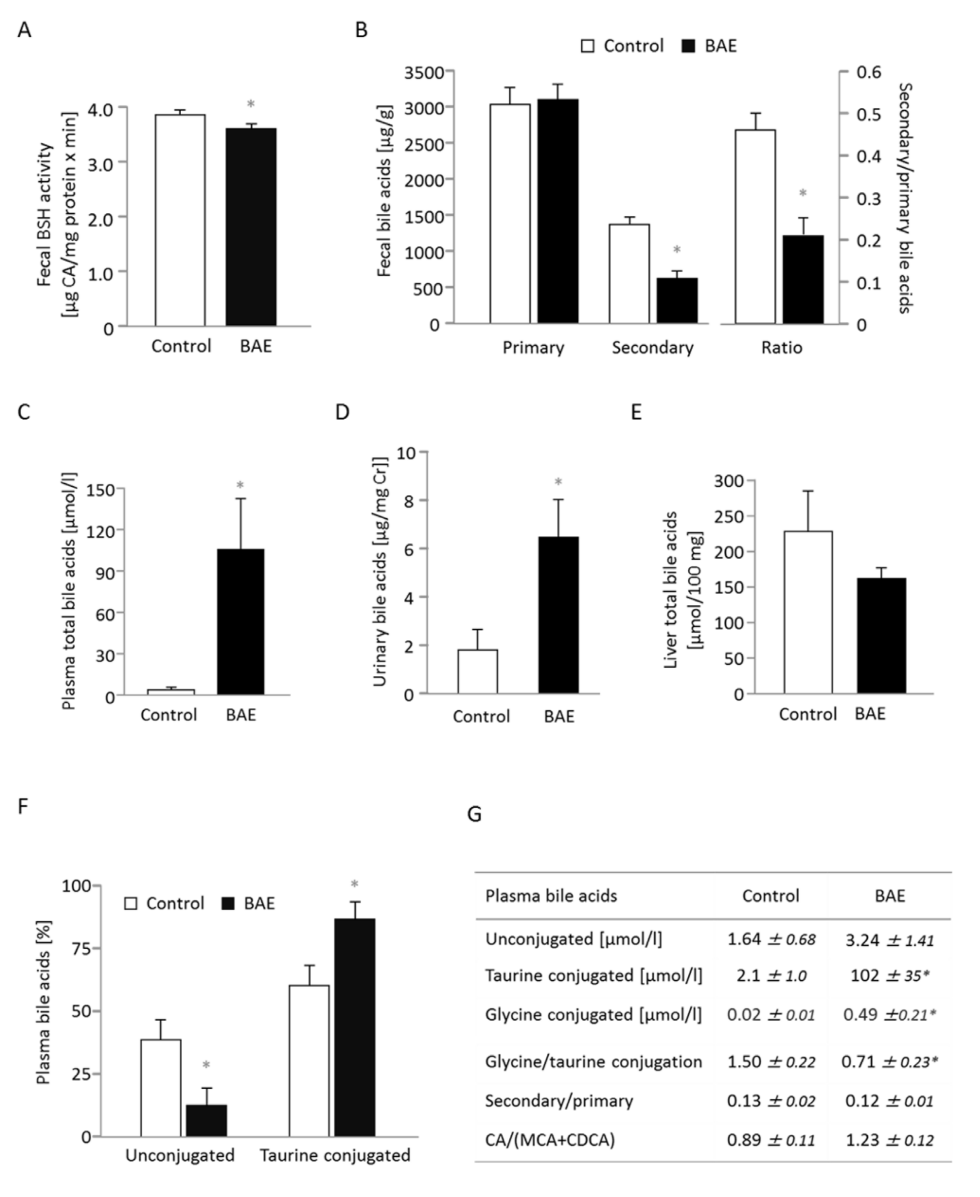
Dietary supplementation of a high fat diet with a brown algae extract (BAE) alters microbial metabolism of bile acids and elevates the concentration of bile acids in systemic circulation of mice Fecal bile salt hydrolase (BSH) activity **A.** as well as concentration of fecal secondary bile acids were significantly reduced in BAE mice, while primary bile acid excretion in feces was similar compared to control mice **B.** The concentration of total bile acids in plasma **C.** and urine **D.** was significantly higher in BAE than control mice, but hepatic bile acids were not different **E.** Urinary bile acid concentration was related to creatinine (Cr) concentration. In plasma, the ratio of conjugated to unconjugated bile acids was significantly modified with a relative decrease of unconjugated bile acids and an increase of glycine and taurine conjugation in the BAE group **F.**, **G.** Furthermore, the ratio of cholic acid (CA) to muricholic (MCA) and chenodeoxycholic acid (CDCA) tended to be higher, whereas the secondary-to-primary bile acid-ratio was not different in BAE mice **G.** Data are means ± SEM (*n* = 6 - 7). Statistically significant differences (*p* < 0.05) are indicated as *.

The addition of BAE into the high-fat diet significantly reduced the activity of the microbial enzyme bile salt hydrolase (BSH) and the concentration of secondary bile acids in feces (Figure 2A, 2B). However, fecal excretion of primary (Figure 2B) and total bile acids (10277 ± 429 vs. 9275 ± 402 µg/g) did not differ between the control and BAE mice. As a result, the ratio of secondary to primary bile acids was 2.2-fold lower for in the BAE group (Figure 2B). These findings indicate that the BAE reduced the capacity of the gut microbiota not only for deconjugation but also for dehydrolyzation of the steroid moiety of bile salts. The BAE fed mice exhibited significantly increased total bile acid levels in systemic plasma and in urine (Figure 2C, 2D); while the levels in liver were not different (Figures 2E) compared to the control mice. The plasma profile of bile acids (Supplementary Figure 1) reveals that all bile acids including cholic acid (CA), muricholic acid (MCA) and chenodeoxycholic acid (CDCA) as well as their derivatives the secondary bile acids deoxycholic acid (DCA), hyodeoxycholic acid (HDCA), lithocholic acid (LCA) and ursodeoxycholic acid (UDCA) were increased upon BAE supplementation. There was however a borderline significant (*p* = 0.061) shift towards the cholic acid pathway indicated by the higher CA/(MCA+CDCA) ratio present in the BAE group (Figure 2G). More importantly, BAE mice showed a 50-fold increase in taurine conjugates plus a 25-fold increase in glycine conjugates along with a 2-fold decrease in unconjugated bile acids in systemic circulation (Figure 2G). Collectively, results indicate that BAE alters drastically bile acid homeostasis by enhancing enterohepatic circulation via modification of microbial metabolism and enhancement of intestinal absorption of total bile acids, but in particular of primary bile acids conjugated with taurine (Figure 2F), without affecting total bile acid excretion in feces.

### The expression of the majority of genes involved in post-absorptive bile acid metabolism are not affected by BAE supplementation

We examined mRNA levels of genes involved in bile acid uptake and binding, export, synthesis and excretion in the ileum, liver and kidney after 4 and 12 h of fasting (Table 2). Because gene expression was very similar for the two fasting periods, mRNA levels are shown only for one time point. In BAE mice, genes involved in bile acid absorption and binding in the ileum were slightly but not significantly higher expressed compared to the control mice. This trend is in line with the BAE-induced increase in circulating bile acids, pointing to enhanced delivery of bile acids to the distal small intestine followed by increased reabsorption into the enterohepatic circulation. Although there was a marginal trend for increased *Ntcp1* mRNA in BAE mice (*p* = 0.057) suggesting a higher hepatic bile acid clearance from the portal vein, there was no apparent effect on genes involved in the canalicular secretion to the biliary duct (*Bsep, Abcg8, Abcc2*), export of bile acids to the circulation (*Abcc3, Abcc4*) or *Fxr* in the liver. Furthermore, the mRNA levels of *Cyp7a1*, which is the rate limiting enzyme in bile acid synthesis, were similar between groups; whereas *Cyp8b1* (synthesis of cholic acid) depicted an upward trend under BAE supplementation (*p* = 0.088). Genes involved in the renal excretion of bile acids showed slight induction in the BAE group with a significant difference in *Abcc2* mRNA levels compared to the control. These results agree with the increased renal excretion of the circulating bile acids in BAE mice.

**Table 2:** Genes involved in bile acid metabolism in the ileum, liver and kidney are only partly modulated in mice fed a high fat diet supplemented with brown algae extract (BAE)

Gene	Function	mRNA level (BAE)
**Ileum**		
*Asbt*	apical membrane, active absorption of mainly conjugated bile acids (enterohepatic circulation/preservation of the bile acid pool)	1.48 *± 0.38*
*Fabp6*	cytosolic bile acid binding after uptake	1.29 *± 0.14*
**Liver**		
*Ntcp1*	basolateral sinusoidal membrane, bile acid clearance from portal blood (enterohepatic circulation/preservation of the bile acid pool)	1.38 *± 0.16*
*Bsep*	canalicular export of bile acids to the bile duct	0.92 *± 0.13*
*Abcg8*	canalicular cholesterol export to the bile duct	1.19 *± 0.15*
*Abcc2*	canalicular bile acid and xenobiotic export to the bile duct	1.13 *± 0.09*
*Abcc3*	export of bile acids to the circulation	0.99 *± 0.07*
*Abcc4*	export of bile acids to the circulation	0.86 *± 0.13*
*Fxr*	bile acid activated transcription factor	0.92 *± 0.08*
*Cyp7a1*	rate limiting first step in major bile acid synthesis	1.13 *± 0.28*
*Cyp8b1*	rate limiting step in cholic acid synthesis	1.68 *± 0.32*
*Cyp7b1*	alternative bile acid synthesis	1.55 *± 0.43*
**Kidney**		
*Asbt*	apical membrane, reabsorption of bile acids from urine	1.18 *± 0.11*
*Abcc2*	apical membrane, excretion of bile acids to the urine	1.19 ± 0.05*
*Abcc4*	apical membrane, excretion of bile acids to the urine	1.22 *± 0.24*

Relative mRNA levels were determined by relating the expression of target to housekeeping genes. Data are means ± SEM (*n* = 6 - 8), expressed in relation to the mean of control mice. Statistically significant (*p* < 0.05) differences are indicated as *.

### BAE modulates substrate oxidation and intestinal expression of genes related to bile acid, glucose and fatty acid metabolism

**Figure 3:**
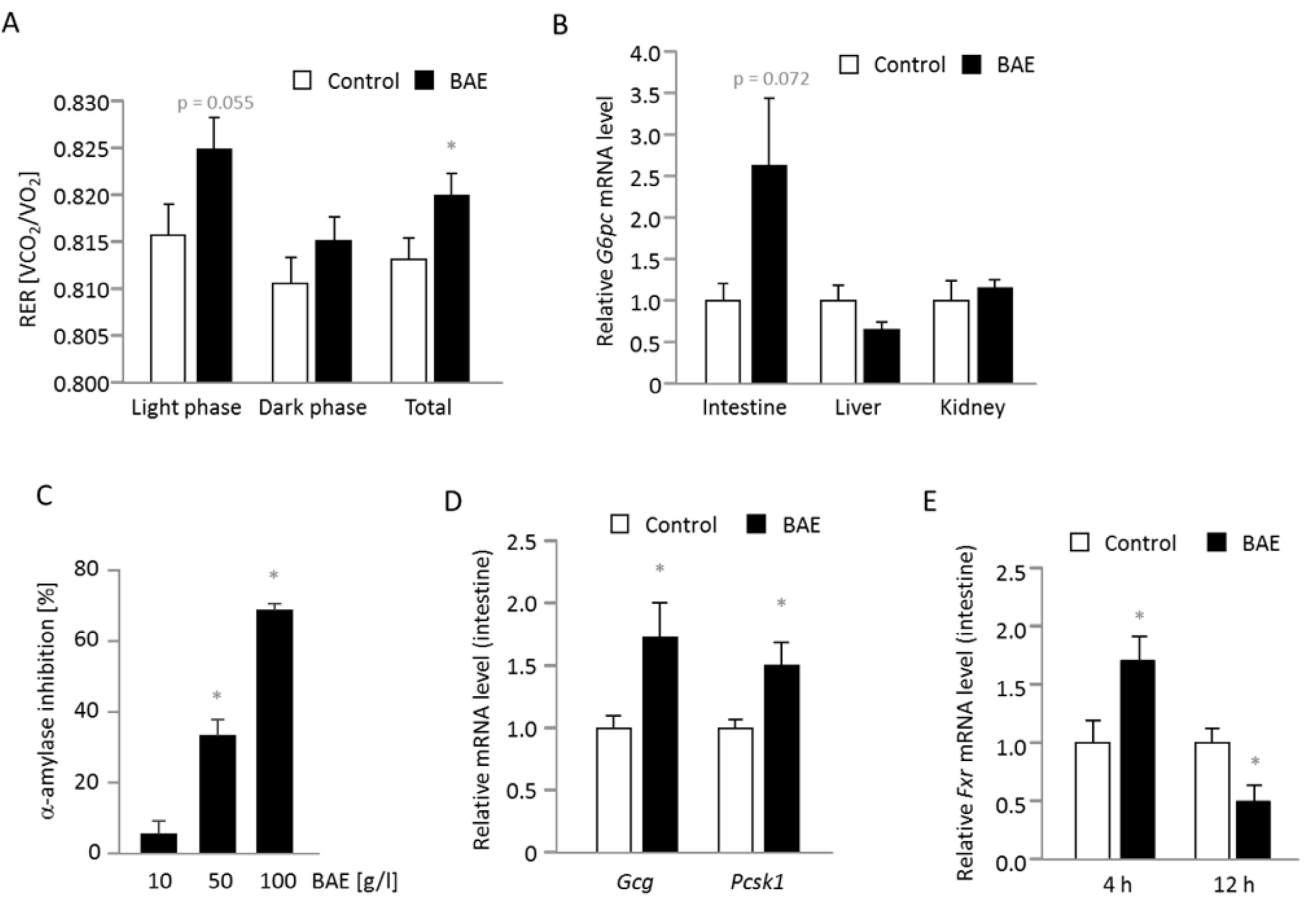
Substrate oxidation, intestinal glucose metabolism and genes involved in glucose and bile acid metabolism are modulated in mice fed a high fat diet supplemented with a brown algae extract (BAE) The respiratory exchange ratio (RER) representing the substrate used for oxidation is significantly higher in BAE mice indicative of increased glucose oxidation especially during the light phase **A.** The mRNA levels of *G6pc* encoding the catalytic unit of the glucose-6-phosphatase that mainly drives gluconeogenesis is higher in the intestine, but not affected in the liver and kidney of 12 h fasted BAE mice **B.** Furthermore, the BAE dose-dependently inhibits the *in vitro* activity of α-amylase suggesting an inhibition of carbohydrate digestion and glucose release **C.** The intestinal mRNA levels of *Gcg* and *Pcsk1* were significantly higher in 4 h fasted BAE mice **D.**, while the expression of *Fxr* was time dependently modulated **E.** Relative mRNA levels were determined by relating the expression of target to housekeeping genes. The mean of the control group was set to be 1. Data are means + SEM (*n* = 7 - 8, except for C, where *n* = 2 - 3). Statistically significant differences (*p* < 0.05) comparing control with BAE mice (except for C, when BAE was compared to water) are indicated as *.

Dietary BAE had no effect on energy expenditure (Supplementary Figure 3), whereas the respiratory exchange ratio was significantly increased compared to the control group (Figure 3A) indicating augmented glucose oxidation especially during the light phase, when the mice were naturally resting. We then examined mRNA levels of genes involved in endogenous gluconeogenesis and found a borderline significant (*p* = 0.072) induction of intestinal *G6pc* mRNA in 12 h fasted BAE mice (Figure 3B), whereas hepatic and renal *G6pc* mRNA levels were either slightly lower or not different compared to the control mice. Interestingly, the effect of BAE on intestinal *G6pc* expression was only observable at 12 but not at 4 h of fasting (1.32 ± 0.33, mean of BAE relative to the mean of control mice). The other key gene of endogenous gluconeogenesis, *Pck1*, was not significantly regulated at the mRNA level in the intestine (ileum: 1.51 ± 0.46; liver: 0.91 ± 0.09; mean of BAE related to the mean of control mice, which was set to be 1). Due to the fact that fasted blood glucose was reduced and intestinal gluconeogenesis appeared to be increased, we continued to determine further factors influencing glucose metabolism. Accordingly, we targeted intestinal glucose release by measuring the activity of α-amylase and found that BAE strongly and dose-dependently inhibits the *in vitro* enzyme activity (Figure 3C). Another intestinal target of BAE could be the intestinal hormone glucagon-like peptide-1 (GLP-1) that is responsible for amplifying insulin secretion and blood glucose clearance in response to nutrient ingestion. GLP-1 is cleaved from its precursor glucagon (*Gcg*) by a prohormone convertase (*Pcsk1*) and rapidly inactivated through degradation by a peptidase encoded by the gene *Dpp4*. In 4 h fasted mice (a time point close to nutrient ingestion) the *Gcg* and *Pcsk1* were significantly induced at the mRNA level in BAE mice (Figure 3D), while *Dpp4* was not significantly modified (1.0 ± 0.1 vs. 1.3 ± 0.2 in control vs. BAE mice). After 12 h however, *Dpp4* mRNA was reduced in BAE mice (1.0 ± 0.3 vs. 0.4 ± 0.1; *p* = 0.073) suggesting that GLP-1 activity may have been higher compared to control mice. In view of the BAE-induced changes in bile acid homeostasis we measured the mRNA level of the bile acid activated transcription factor FXR and observed a time dependent modulation (Figure 3E). Compared to the control, BAE mice exhibited significantly higher *Fxr* mRNA levels after 4 h and a significantly lower abundance of after 12 h of fasting.

### Life span and activation of AMPK and mTOR are not significantly modified by BAE

**Figure 4:**
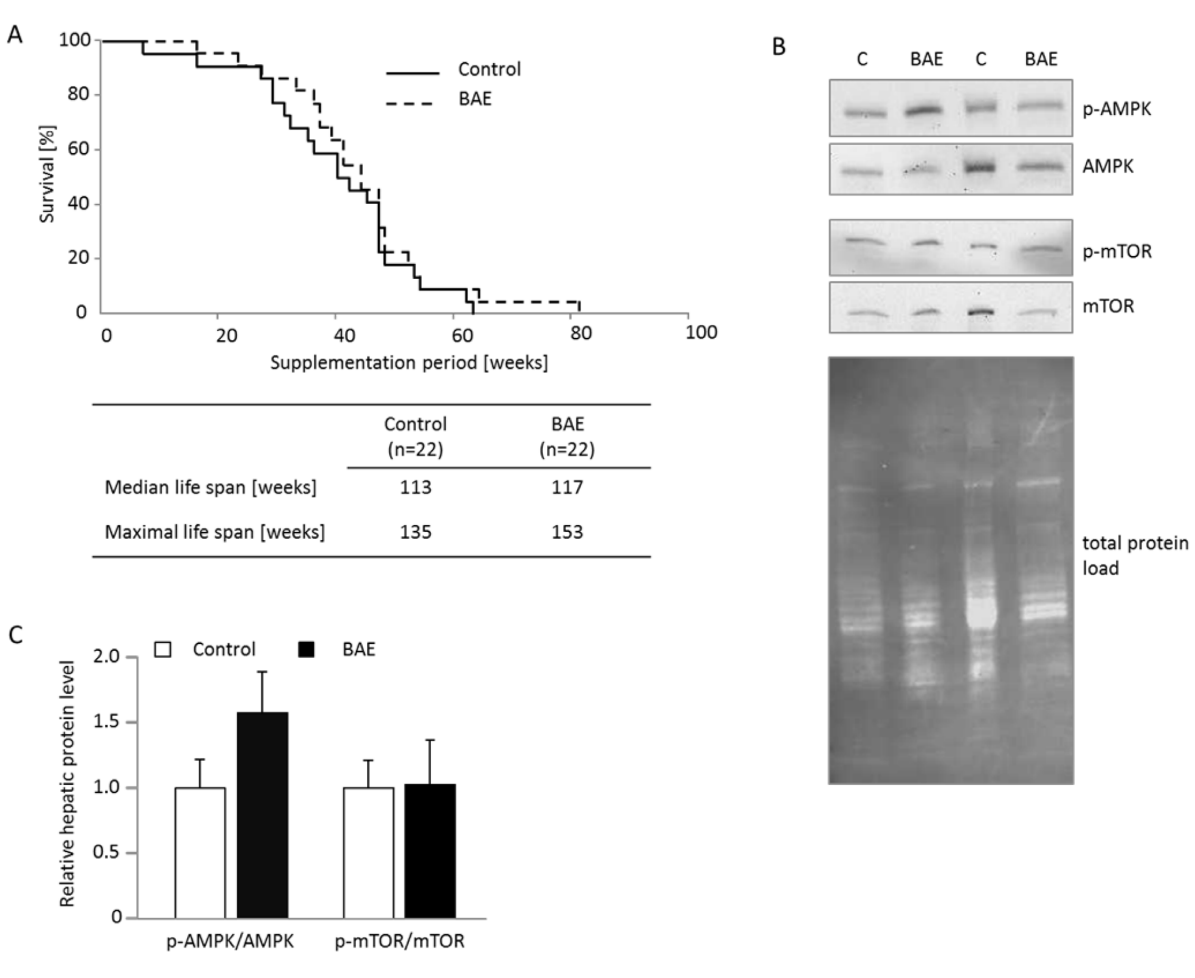
Brown algae extract (BAE) supplementation showed no significant effect on life span and activation of the key ageing pathway mTOR, but tended to increase AMPK signaling in the liver of mice fed a high fat diet Survival of 18 months old mice placed on the high fat diet with half of them receiving dietary BAE supplementation was assessed. The median and maximal life spans were not significantly different between BAE and control mice **A.** and likewise no activation of mTOR (p-mTOR/mTOR) was evident **B.** However, AMPK tended to be activated in BAE mice determined as the ratio of phosphorylated AMPK (p-AMPK) to total AMPK. Cropped Western blot images visualizing the respective target bands show representative animals of the groups. Total protein load per lane was used for target protein band normalization. The relative protein band intensity was densitometrically analyzed for of all animals of the group, individual ratios calculated and the means + SEM (*n* = 6 - 7) per group are given in **C.**

Since BAE mice showed an improved metabolic health, we determined the survival of 18 months old mice that were introduced to the high fat diet in comparison to mice that were additionally supplemented with BAE. Although statistically not significant, maximal life span of BAE mice was 13 % higher and the survival curve slightly shifted rightwards compared to age-matched control mice (Figure 4A). These results also illustrate the good tolerance of dietary BAE in the mice. Furthermore, the analysis of the degree of phosphorylation of key proteins involved in nutrient sensing in the liver resulted in a non-significant 1.6-fold induction of the p-AMPK/AMPK and no apparent change of the p-mTOR/mTOR ratio in BAE mice (Figure 4B).

## DISCUSSION

C57Bl/6 mice fed a HFD develop massive obesity related pathologies including fat deposition in various organs, altered plasma lipids and impaired insulin sensitivity. Supplementation with different brown algae has been shown to improve these HFD related pathologies [15, 17, 26] and the authors explained such beneficial effects by potential anti-adipogenic effects referring to cell culture experiments in which brown algae exhibited a potent inhibition of adipocyte differentiation [8, 17, 27]. However, it remains questionable whether potentially bioactive brown algae compounds are able to reach the circulation and adipose tissue in relevant amounts without prior hepatic biotransformation. It may be rather likely that the metabolic benefit from dietary BAE supplementation is a consequence of primary events occurring in the gut. Therefore we mainly focused on intestinal processes to elucidate underlying mechanisms and pathways mediating the metabolic benefits from the long term dietary supplementation with our BAE. In addition to confirm BAE-induced loss of body weight and fat mass as well as normalization of blood glucose, we uncovered a multi-factorial mechanism that involves (1) profound alterations in bile acid homeostasis and signaling in the intestine, (2) changes in intestinal and systemic glucose metabolism likely including increased intestinal gluconeogenesis, (3) supposedly increased GLP-1 via increased *Gcg* and *Pcsk1* and reduced *Dpp4* expression possibly contributing to promote systemic insulin sensitivity, and (4) a dose dependent inhibition of α-amylase activity expectably limiting carbohydrate digestion and glucose release in the intestinal lumen. We moreover propose that the beneficial metabolic effects of our BAE are at least partly mediated by alterations in gut microbiota associated with fermentation of indigestible polysaccharides that are major components of brown algae such as alginates and fucoidans.

The BAE used in our study contained 12 % fiber, which enhanced the fiber content of the control diet from 5.8 to 6.4. The high water binding capacity of algal fibers, especially alginates, increases the stool volume and reduces the transit time of the intestinal content likely explaining the higher fecal mass (fecal dry weight and water content) observed in the BAE group. Furthermore, alginates are known to form a viscous gel that slows down intestinal glucose and fat absorption and reduces the postprandial peaks of glucose, insulin, and lipids [28]. Although most algal polysaccharides are soluble, they are supposed to be poorly fermented by intestinal bacteria with only few exceptions [29-31]. However, these observations may be subjected to some limitation since they were made under *in vitro* conditions. For instance, mice are known to exhibit coprophagy that would augment the digestibility of dietary components and the energy harvest from rather slowly fermented fibers. Furthermore, it is also reasonable to expect a certain degree of fermentation *in vivo* of brown algal polysaccharides involving alternative pathways of alginate degradation [30]. These observations may explain why we did not observe differences in fecal energy density, suggesting that dietary energy utilization was similar between groups despite different dietary fiber concentrations. This result also indicates that fecal energy losses could be excluded as reason for the lower body weight and fat mass in BAE mice, but rather point to other mechanisms such as metabolic signaling triggered by microbial products of polysaccharide fermentation in the gut.

Fermentation of undigested polysaccharides in the large intestine shapes the microbiota profile such that bacteria that promote leanness of the host are supported while the growth of metabolically adverse phyla is contained. Accordingly, a diet rich in protein or fat like our HFD shifts the microbiome to a profile that is correlated with an adverse metabolic phenotype promoting fat deposition and insulin resistance in the host, which have been shown to be normalized through the increase of dietary fibers [32]. In addition, consumption of HFD increases the flow of bile acids into the intestine to support fat digestion. In turn, increased concentration of bile acids in the gut stimulates proliferation of bacteria that are associated with production of toxic secondary bile acids, inflammation and obesity [33, 34]. Interestingly, algal polysaccharides have repeatedly been shown to increase the abundance of what is considered beneficial bacteria such as bifidobacteria [28]. In our study, such changes in microbial composition along with the BAE-enhanced enteral supply of fiber should have promoted the production of short chain fatty (SCFA), including acetate, propionate, and butyrate [35]. SCFA can be used for *de novo* synthesis of glucose and lipids, but also appear to act as metabolic signaling molecules regulating energy homeostasis through activation of the G-protein coupled receptors GPR43 and 41 [36-38]. Furthermore, butyrate and propionate induce intestinal gluconeogenesis, which has been suggested to pivotally mediate the metabolic improvement attributed to dietary fibers [39, 40]. SCFA activate intestinal gluconeogenesis via induction of gluconeogenic genes such as *G6pc* and tissue allocation of energy and gluconeogenic substrates [40]. In contrast to hepatic gluconeogenesis, which is supposed to contribute to insulin resistance, glucose newly synthesized by the intestinal epithelium is released to the portal vein, where it is sensed by vagal nerves and promotes normalization of impaired systemic glucose metabolism [39-41]. The expression of *G6pc* was increased in the intestine but not in the liver of BAE mice, indicating a potential increase of intestinal gluconeogenesis upon BAE supplementation. Therefore we hypothesize that fermentation of BAE indigestible polysaccharides and their degradation to SCFA induced intestinal gluconeogenesis and thereby contributed to ameliorate the impairment of glucose metabolism otherwise caused by the feeding of HFD to mice. Similar results were obtained from fructo-oligosaccharide supplemented mice, where diet-induced obesity and related insulin resistance were attenuated in wild type but not knock-out mice with deletion of the intestinal *G6pc* [40].

The metabolic dysfunction associated with obesity also involves failure in the modulation of substrate oxidation in response to fasting or feeding. The absence of switching from fatty acid to glucose oxidation is called metabolic inflexibility and is believed to be caused by reduced insulin sensitivity, hepatic liver accumulation and impairment of hepatic glycolysis [42]. Metabolic flexibility was also impaired in high fat diet-induced obese rats and mice [43, 44], but could be restored by feeding diets enriched in viscous fibers or proteins. Compared to their obese litter mates, fiber-fed rats showed an expedited transition from fatty acid to glucose oxidation during fasting [44]. One possible explanation may be the induction of intestinal gluconeogenesis that is promoted by dietary fibers and protein [41]. We have similarly observed that BAE-fed mice compared to control counterparts show a significantly higher respiratory exchange ratio indicative of higher glucose oxidation especially during resting in the light phase. This result suggests an improvement of metabolic flexibility in BAE-fed mice similar to rats fed viscous fiber and emphasize the role that viscous fibers appear to play in the mechanism underlying the beneficial effects of BAE.

Further metabolic alterations entailed by HFD feeding involve changes in bile acid homeostasis, which include reduction of bile acids in systemic circulation [3, 45] along with increased flow of bile acids into the gut followed by fecal excretion [33]. In addition, postprandial bile acid response in the circulation is lower compared to normal weight controls [46]. Surprisingly, the total level of circulating bile acids was dramatically increased in the BAE supplemented group, whereas their fecal output remained unaltered. Furthermore, BAE selectively enhanced the proportion of conjugated bile acids while decreasing the percentage of unconjugated species in systemic circulation. Similarly, gastric bypass surgery, one of the most effective interventions against obesity, is known to enhance the circulating pool of bile acids dominated by conjugated primary forms [47]. We suppose that dietary BAE supplementation protects conjugated primary bile acids and reduces the formation of secondary ones in the intestine via partial inhibition of both BSH mediated deamidation and subsequent dehydroxylation. Furthermore, the BAE-induced decline in BSH activity also may have prevented bile acid accumulation in the gut and subsequent fecal excretion, because conjugated bile acids are absorbed more efficiently than their unconjugated counterparts [35]. In the human intestine, BSH activity is primarily contributed by bacteria of the genera *Lactobacillus* and *Clostridium*, whereas the 7α-dehydroxylating capacity is mainly provided by a small group of bacteria belonging to the Clostridium cluster XIVa [33]. A growing number of studies link the intestinal predominance of such bacteria to host adiposity, inflammation and liver disease [33, 48]. Therefore, the improvement in the metabolic phenotype of HFD-fed mice in concert with the decline in intestinal deconjugation and dehydroxylation of bile acids must be associated with a remodeling of the gut microbial ecology driven by the feeding of BAE. Although the role of circulating bile acids in the fasted state remains to be elucidated, mounting evidence indicates that they exert beneficial metabolic functions. In this context, it was shown that dietary bile acids [49] and activation of the bile acid receptors TGR5 in adipose tissue and FXR in intestine but not in liver [45, 50, 51] increases secretion of GLP-1, insulin sensitivity, energy expenditure, UCP1 activity and browning of white adipose tissue, thereby counteracting obesity. The supplementation of the HFD with BAE amplified intestinal but not hepatic FXR expression, suggesting that alterations in bile acid signaling in the gut played a pivotal role in mediating its anti-obesity action. In addition, induction of *Gcg* and *Pcsk1* and repression of *Dpp4* expression have likely contributed to the expected bile acid-induced release of GLP-1. Enhanced GLP-1 production and reduction of Dpp4 may have improved insulin sensitivity and normalize blood glucose through increased stability of GLP-1 similar to pharmacological Dpp4 inhibition [52].

Our BAE also contained secondary metabolites with potential bioactivity such as dieckol, eckol and other ploroglucinol derivatives, ergosterol, gallic acid and fucoxanthin (Supplementary Table 1). Especially the phloroglucinol derivatives (so-called phlorotannins) and fucoxanthin have been observed to normalize blood glucose and triglycerides, inhibit adipogenesis, activate UCP1 and increase energy expenditure in cultured cells and diet induced obese mice [16, 18, 19, 53-56]. It may therefore be possible that these algal compounds also contribute to the beneficial metabolic effects in our BAE fed mice. However, it is noteworthy that we did not observe any changes in energy expenditure, expression of UCP1 in adipose tissue or proteins involved in skeletal muscle function in BAE compared to control mice (Supplementary Data). This may be explained by the concentration and bioavailability of algal bioactives that were too low and the animal model and experimental setup that possibly were unsuitable or not sensitive enough to display changes in thermogenesis and energy expenditure.

We likewise did not observe any significant differences in median and maximal life span indicating that ageing and fitness of the mice were not affected. In line with that the hepatic expression and activation of mTOR, which has been identified as important ageing related pathway [57], was similar in the BAE and the control group. This is an unexpected finding as a metabolic improvement like the one observed in our BAE mice could have qualified BAE supplementation as a dietary intervention that mimics caloric restriction potentially delaying ageing processes. However, this assumption may be considered as refuted based on our results. Furthermore, the energy sensing signaling pathway AMPK has been suggested a molecular target of brown algal compounds in the literature [8, 9], yet only tended to be induced upon BAE supplementation in our mice. Taken together, we do not find any explicit effects on cellular ageing in diet induced obese mice supplemented with BAE despite apparent phenotypic and metabolic improvement.

We hypothesize that the reduced body weight gain and fat mass as well as the improved glucose metabolism depicted by mice fed a HFD supplemented with BAE were mainly driven by the high content of algal polysaccharides in BAE. On the one hand, they form a viscous gel in the intestine that appears to inhibit digestive enzyme activity, slow down absorption and postprandial peak of carbohydrates and lipids, retard transit time of digesta, and protect primary bile acids against microbial metabolism increasing in this manner their delivery to and absorption from the distal small intestine. On the other hand, the increased production of SCFA that is expected from fermentation of BAE polysaccharides in the gut improves the metabolic phenotype by normalizing blood glucose, and increasing intestinal gluconeogenesis as well as metabolic flexibility. Furthermore, through the supply of fermentable polysaccharides the growth of beneficial bacteria is likely promoted, shifting the gut microbial composition to a profile that promotes leanness possibly via alteration of bile acid metabolism and signaling.

## MATERIALS AND METHODS

Animal experiments were performed according to German and international regulations of animal welfare. The experimental protocol was approved by the local authority (Ministry of Energy, Agriculture, the Environment and Rural Areas, Schleswig-Holstein). Sixteen male C57BL/6J mice were purchased from Janvier (Saint Berthevin Cedex, France) at the age of 6-8 weeks. Mice were housed individually in macrolon cages with environmental enrichment under controlled climatic conditions (55 % relative humidity, 22-24 °C and 12 h light/dark cycle). All animals had free access to drinking water and the experimental diet. The purified semi-synthetic experimental diet was high in fat and sugar content (a so-called Western type diet) and was composed of (%): sucrose, 32.8; butter fat, 21.2; casein, 17.1; maize starch, 14.5; and cholesterol, 1.25 (Ssniff special diets, Soest, Germany). For simplicity, the diet will be referred to as ‘high fat diet’ in the following, despite the fact that it is actually a high calorie diet. Next to the control group, there was the supplementation group that received the identical experimental diet (high fat diet) supplemented with 5 % of a freeze-dried aqueous extract of the Atlantic brown algae *Saccorhiza polyschides* (CRM, Coastal Research & Management, Kiel, Germany) (BAE). The major secondary metabolites of BAE are dieckol, eckol, bieckol, other polyphenols, ergosterol and fucoxanthin as determined by LC-quadrupole time of flight mass spectrometer analysis (Supplementary Methods and Supplementary Table 2). The control and BAE diet differed in their energy (21 vs. 19.8 MJ/kg) and fiber (5.8 vs. 6.4 %) content. Fiber content was determined by a standard analytical method (§ 64 German Food and Feed Code L00.00-18) as non-enzymatically hydrolysable polysaccharides.

During the entire feeding period the health conditions of the mice were controlled daily. Food intake and body weight were determined daily and weekly, respectively. After 32 weeks, 4 h and 12 h fasted mice were anaesthetized with CO_2_ and killed by cervical dislocation. Liver, interscapular brown adipose tissue (BAT) and parts of small intestine (ileum) were removed and stored at -80 °C until analysis. Additionally, parts of liver and epididymal visceral adipose tissue (WAT) were put in RNAlater (Qiagen, Hilden, Germany)™ and kept at -20 °C until RNA isolation. Blood was collected in heparinized tubes, and centrifuged (3000g, 4 °C, 10min) to obtain plasma. Plasma samples were stored at -80 °C until usage.

### Body composition by nuclear magnetic resonance (NMR) and micro-computed tomography (micro-CT)

Time domain NMR was used to determine lean and fat mass in all animals using the benchtop NMR analyzer Minispec LF90 (Bruker Biospin MRI GmbH, Ettlingen Germany). This is a rapid non-invasive examination of whole body fat tissue, lean tissue and free fluid based on the response to various radiofrequency pulse sequences. Due to the short time of measurement and the easy handling, there is no need to anesthetize the mice, allowing the minimization of stress to the animals. For detection of abdominal adiposity micro-CT was applied. Four randomly assigned mice per group were anesthetized with Ketamine/Xylazine and the abdominal region between the first and the fifth lumbar vertebra was scanned using a cone-beam in vivo micro-computed tomography system (vivaCT 40 Scanner, Scanco Inc., Brüttisellen, Suisse). The scan was performed using the following parameters: energy settings of the X-ray source 45 kVp and 177 µA, voxel size 76 µm, integration time 300 ms, 250 projections per 180°. Visceral and subcutaneous fat volumes were calculated as described previously [6] using a Canny Edge detection.

### Energy expenditure (EE) by indirect calorimetry

EE of four randomly assigned mice per group was assessed by indirect calorimetry measuring the volumes of O_2_ consumption (VO_2_) and CO_2_ production (VCO_2_) using the TSE PhenoMaster (TSE Systems GmbH, Bad Homburg, Germany). Mice were placed in respiratory chambers for 48 h (including 24 h of adaptation) with an air flow of 0.35 l/min and air sampling every 15 min for O2 and CO2 analysis. EE was calculated as follows: EE = (3.941*VO2 + 1.106*VCO2)*4.1868/1000 and expressed as kJ/h*kg^0.75^.

### Plasma analyses

Plasma concentrations of leptin (R&D Systems, Abingdon, UK), adiponectin (Abcam, Cambridge, UK) and insulin (Merck Millipore, Darmstadt, Germany) were measured using commercially available ELISA kits. Fasted plasma concentration of glucose was determined with a colorimetric assay (Cayman, Ann Arbor, USA) using enzymatic oxidation of glucose to δ-gluconolactone by FAD-dependent reduction of glucose oxidase and subsequent production of hydrogen peroxide. Total triglycerides and cholesterol were measured with commercially available enzymatic activity based colorimetric assays (Fluitest^®^, Analyticon, Lichtenfels, Germany) according to the manufacturer’s protocol. Activity of the hepatic transaminases ALT and AST in the plasma were determined with commercially available colorimetric assay kits (Sigma-Aldrich, Steinheim, Germany) and performed according to the manufacturer’s instruction.

### Fecal and urinary sample collection and analyses

For fecal and urine sample collection, the mice were placed in metabolic cages over a period of one week. Feces and urine were collected daily, pooled over the week and stored at -40 °C until analyses. Food intake was recorded daily. Fecal samples were dried for 48 h at 103 °C and grounded. Fecal caloric value was determined with a bomb calorimeter (C 7000, cooler C7002, oxygen filling station C48, IKA^®^-Werke GmbH & Co. KG, Staufen, Germany) and apparent food digestibility was calculated as (total energy intake - fecal energy excretion)/total energy intake*100. Fecal bile acid hydrolase (BSH) activity is a measure of the intestinal capacity for bile acid deconjugation and was determined as previously described [48] with minor modifications. In brief, total protein was isolated from fecal samples, incubated at a concentration of 100 µg/ml with the substrate TCA-d5 sodium salt and BSH activity calculated as µg released CA/mg protein*min.

### Bile acid analysis

The concentration of bile acids in plasma and liver was analyzed by UHPLC-MS/MS. The system consisted of an UPLC-H class (Waters, UK) coupled to a Xevo TQ-S triple quadruple mass spectrometer (Waters, UK). Electrospray ionization was performed in the negative ionization mode. Chromatographic separation was performed on a BEH C18 column (2.1x100 mm, 1.7 µm). The mobile phase consisted of water containing 0.1 % formic acid and 5 mM ammonium acetate (Eluent A) and acetonitrile (Eluent B). Analytes were separated by a gradient elution. The injection volume was 5 µL and the column was maintained at 40 °C. Detection of the bile acids and their glycine and taurine conjugates was performed in the selected reaction monitoring mode. All standards as well as the deuterated internal standards substance (d4-CA, d4-GCA, and d4-GCDCA) were purchased from Sigma-Aldrich (Taufkirchen, Germany) and Steraloids (Newport, USA). The taurine conjugated α-MCA and β-MCA were confirmed by standards but not separated on UPLC column and hence regarded as T-MCA. The standards for glycine conjugated α-MCA and β-MCA were commercially not available and postulated from m/z, retention time and fragmentation as G-MCA. The concentration of bile acids in urine and feces samples was analyzed by UHPLC-MS. In this case, the system consisted of an UPLC-H I-Class (Waters, UK) coupled to a Xevo-G2 QTof mass spectrometer (Waters, UK). Electrospray ionization was performed in the negative ionization mode. Chromatographic separation was performed on a BEH C18 column (2.1 x 100 mm, 1.7 µm). The mobile phase consisted of water (Eluent A) and acetonitrile (Eluent B) both containing 0.1 % formic acid. Analytes were separated by a linear gradient elution. The injection volume was 2 µL and the column was maintained at 40 °C. Detection of the bile acids and their glycine and taurine conjugates was performed by exact mass (+/- 0.01 Da). All standards as well as the deuterated internal standard substance (d4-CDCA) were purchased from Steraloids (Newport, USA).

### RNA isolation and qRT-PCR

Total hepatic RNA was isolated from tissue stored in RNAlater using the Qiagen RNAeasy Mini Kit (Qiagen, Hilden, Germany). Total RNA from mouse brown adipose tissue was isolated according to manufacturer’s instructions, using the PARIS™ kit (Ambion, Kassel, Germany) for parallel isolation of RNA and proteins. Isolation of total RNA from the small intestine was performed with peqGOLD TriFast™ (PEQLAB Biotechnologie GmbH, Erlangen, Germany) following manufacturer’s instructions. RT-PCR primers (Supplementary Table 3) were designed using PRIMER v. 3 input software (v. 0.4.0) or taken from http://pga.mgh.harvard.edu/primerbank/, respectively). Gene expression levels in liver and brown adipose tissues were determined by one-step quantitative reverse transcriptase PCR was performed using the SensiFAST™ SYBR^®^ No-ROX One-Step Kit (Bioline, Luckenwalde, Germany) with SybrGreen detection using a Rotorgene 6000 cycler (Corbett Life Science, Sydney, Australia). Intestinal gene expression levels were also assessed by two-step real-time PCR. Following reverse transcription (Tetro cDNA Synthesis Kit, Bioline, Luckenwalde, Germany) in a thermocycler (Biometra, Göttingen, Germany), cDNA was applied to real-time PCR using SensiFAST™ SYBR^®^ No-ROX Kit (Bioline, Luckenwalde, Germany) in the Rotorgene 6000 cycler. All qRT-PCR data are expressed as relative mRNA levels by dividing individual target gene expression to individual housekeeping gene expression and subsequently relating the individual results of the BAE group to the mean of the control group.

### Western blot analysis

Tissue whole cell protein lysates were prepared with RIPA buffer and the Western blot analysis was performed as previously described in detail [6]. In brief, proteins were separated by SDS-PAGE and transferred onto a PVDF membrane. Target proteins were identified using specific primary antibodies [6, 58] and corresponding secondary antibodies (Abcam, Cambridge, UK). Protein bands were visualized with ECL reagents (Fisher Scientific, Schwerte, Germany) in a ChemiDoc XRS system (BioRad, Munich, Germany). Target protein expression was related to the total protein load per lane, which was assessed by UV induced reaction of the trihalo compounds of Criterion™ TGX Stain-Free™ Precast Gels (BioRad, Munich, Germany) with tryptophane residues of the loaded protein measured as PVDF membrane fluorescence after the transfer of protein from gel to membrane. Afterwards, the ratio of phosphorylated to total target protein (AMPK, mTOR) was calculated for individual animals prior to group mean calculation.

### Life span

For the evaluation of BAE effects on the life span of our mice, 44 male C57BL/6J mice were purchased from Janvier at the age of 18 months. After two weeks of acclimatization on the experimental control diet, the mice were ranked according to their body weight and assigned to either the control or the BAE group paired by body weight means. The animals had free access to the diet and drinking water. The health conditions were controlled daily, food intake and body weight were determined weekly. Mice that suffered from obvious health limitations (e.g. apparent weight loss, sudden and prolonged lethargy, tumours) other than normal during the aging process were excluded. Remaining animals were kept until they died from natural causes.

### Statistical analysis

Statistical analyses comparing the control and BAE group were performed using SPSS version 19.0 software (SPSS Inc.). Kolmogorov-Smirnov and Shapiro-Wilk tests were used to test the data for normal distribution. Data following a Gaussian distribution were analyzed by Student’s t-test. In the case of a non-Gaussian distribution, a Mann-Whitney U-test was performed. Results are expressed as means and SEM, and differences were considered significant when the *p* value was < 0.05 and borderline significant at *p* < 0.1. Kaplan-Meier survival curves were generated with a subsequent log-rank test to compare survival curves of the two groups.

## SUPPLEMENTARY MATERIALS FIGURES AND TABLES





## Abbreviations

BAE: brown algae extract
BAT: brown adipose tissue
BSH: bile salt hydrolase
CA: cholic acid
CDCA: chenodeoxycholic acid
Cr: creatinine
DCA: deoxycholic acid
EE: energy expenditure
HDCA: hyodeoxycholic acid
HFD: high fat diet
LCA: lithocholic acid
MCA: muricholic acid
micro-CT: micro-computed tomography
NMR: nuclear magnetic resonance
RER: respiratory exchange ratio
SCFA: short chain fatty acids
UDCA: ursodeoxycholic acid
WAT: white adipose tissue
