# Supplementary material for: An extract from the Atlantic brown algae Saccorhiza polyschides counteracts diet-induced obesity in mice via a gut related multi-factorial mechanisms
Source: Oncotarget. 2017 May 23;8(43):73501–15. doi: 10.18632/oncotarget.18113 (PMC5650277; doi:10.18632/oncotarget.18113)
Supplement: Supplementary file 1 [file oncotarget-08-73501-s001.pdf]

An extract from the Atlantic brown algae *Saccorhiza polyschides* counteracts diet-induced obesity in mice via a gut related multi-factorial mechanisms

SUPPLEMENTARY MATERIALS

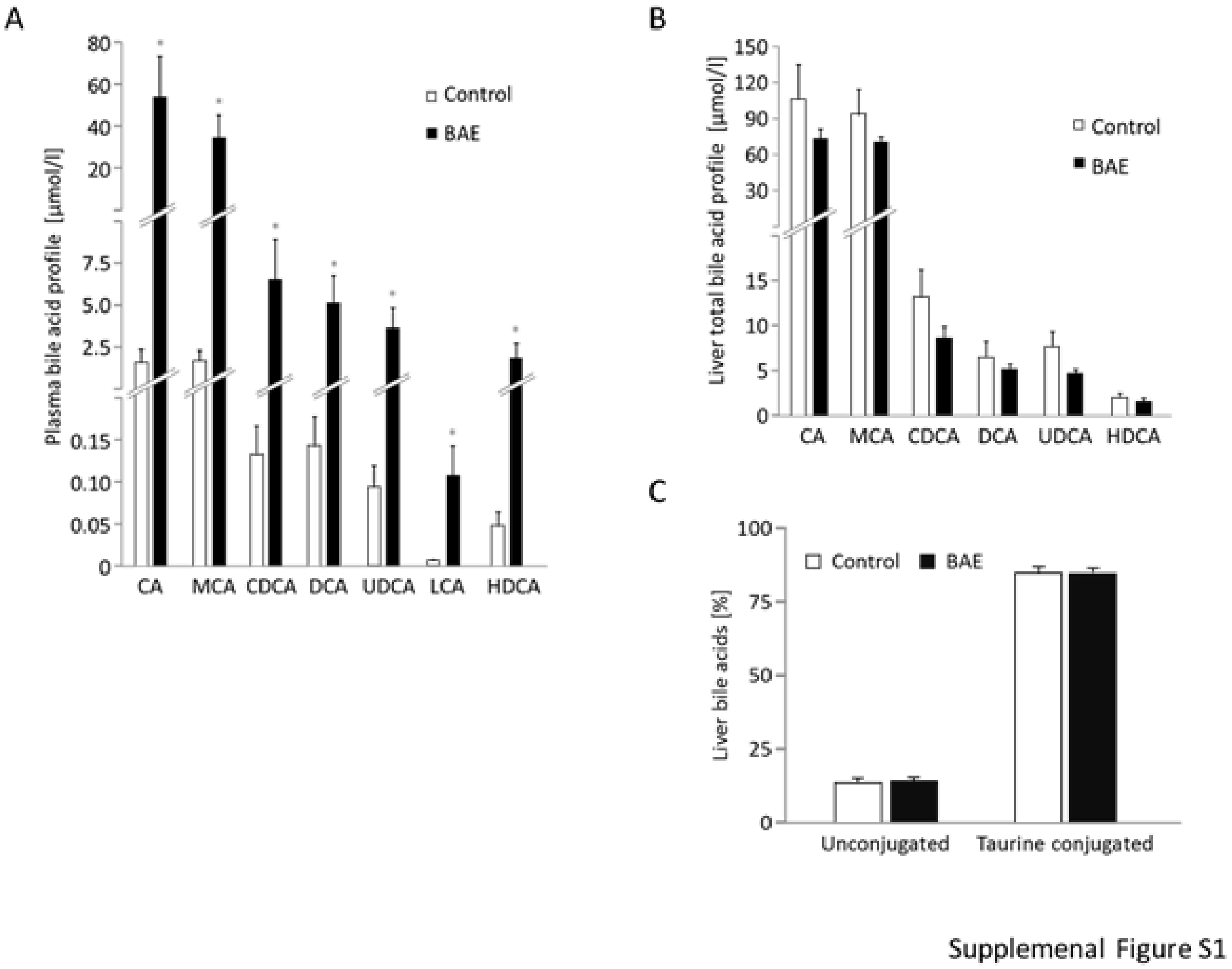

Supplementary Figure 1: Concentration of bile acids in the systemic circulation (A) and liver (B) in mice fed a high fat diet (control) and a high fat diet supplemented with a brown algae extract (BAE). Relative levels of taurine conjugated and unconjugated bile acids were similar between the groups (C). Data are means ± SEM (n=6-7). Statistically significant differences ($p<0.05$) are indicated as *. CA, cholic acid; MCA, muricholic; CDCA, chenodeoxycholic acid; DCA, deoxycholic acid; UDCA, ursodeoxycholic acid; LCA, lithocholic acid; HDCA, hyodeoxycholic acid

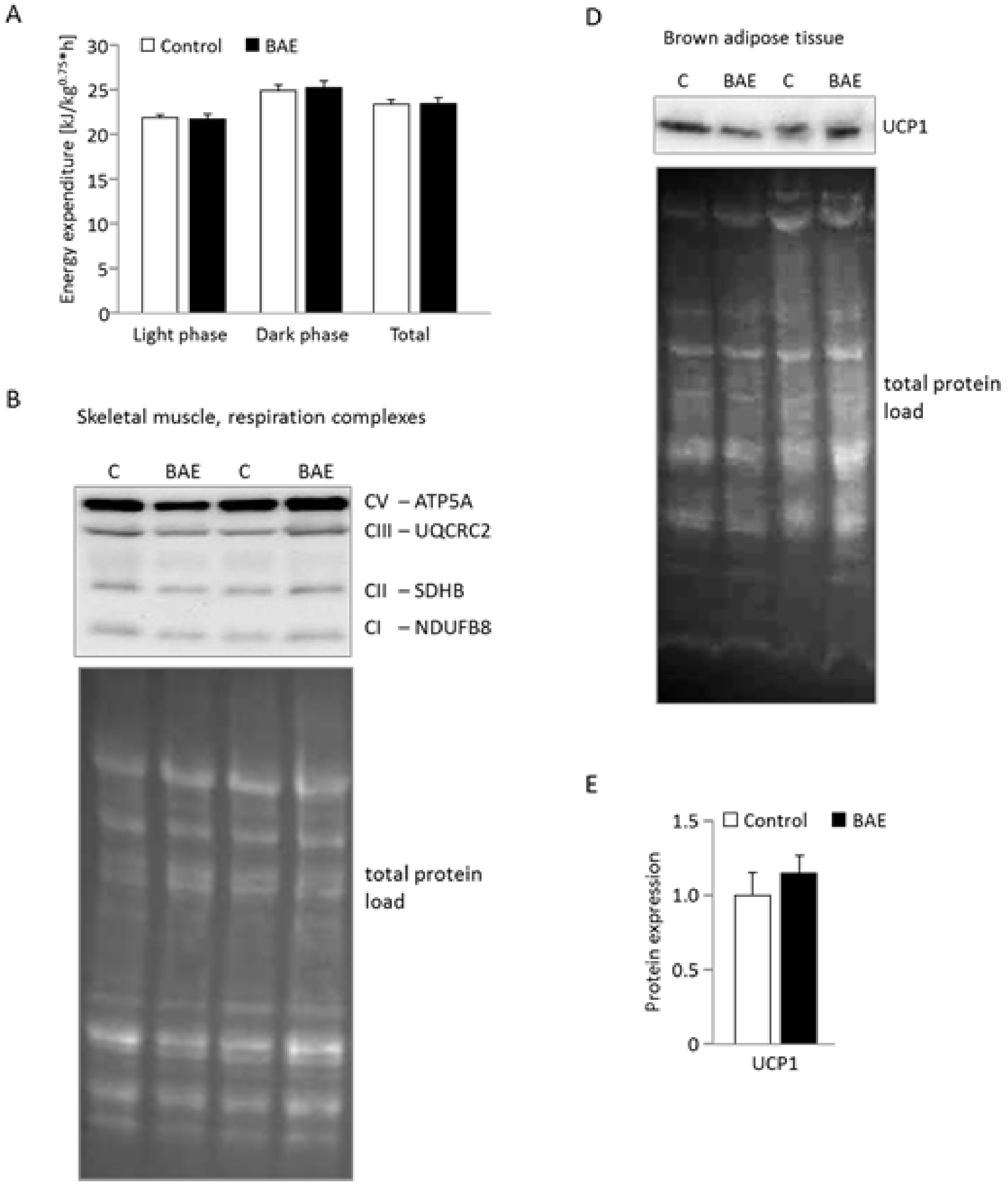

C

	CI NDUFB8	CII SDHB	CIII UQCRC2	CV ATP5A
Protein expression (BAE)	0.70 ±0.15	0.82 ±0.13	0.90 ±0.20	1.14 ±0.16

Supplemental Figure S2

Supplementary Figure 2: Energy expenditure (A) and protein level of OXPHOS proteins in the skeletal muscle (B, C) and uncoupling protein UCP1 in brown adipose tissue (D, E) are not different in mice fed a high fat diet supplemented with a brown algae extract (BAE). Cropped Western blot images visualizing the respective target bands show representative animals of the groups (B, D). Total protein load per lane was used for normalization of target protein band intensity. The relative protein band intensity of all animals per group is depicted in the densitometric analysis data (C, E). The mean of the protein level of the control group was set to be 1; data of the BAE group are related to the control group. Data are means ± SEM (n=7 for A, n=6-7 for C, E). CI-III, V, complexes I-III, V of mitochondrial oxidative phosphorylation (OXPHOS), C, control group

METHODS

LC-MS analysis of the BAE

Chromatographic separation of samples (5 µl injection volume) were conducted on an Acquity BEH Amide HILIC column (2.1 * 100mm, 1.7 µm particles (Waters, Eschborn, Germany) at 30 °C using an UHPLC system (Infinity 1260, Agilent, Waldbronn, Germany). The eluents consisted of 0.1 % formic acid (A) and acetonitrile containing 0.1 % formic acid (B). The gradient with a flow rate of 0.25 ml / min started with 95 % B (0.5 min) and decreased to 40 % within 7 min, kept constant for 1 min, followed by an increase to 95 % B (1 min) and a regeneration step for 5.5 min. Detection was conducted by quadrupole time-of-flight mass spectrometer ESI-QTOF-MS (microtof-Q II, Bruker, Bremen, Germany) using electrospray ionization (positive [M+H] and negative [M-H] ionization mode). The operation conditions were as follows: dry gas temperature at 210 °C, flow rate of 6 L/min; nebulizer gas pressure 1 bar; mass range 75 to 1000 m/z with an optimimum range between 100 to 300 m/z (z = 1); spectra acquisition rate 4 Hz. For calibration a premixed lithium-formate solution (Sigma-Aldrich, Taufkirchen,Germany) was used. The data were analysed by the software DataAnalysis 4.3 (Bruker, Bremen, Germany).

For Supplementary Tables see in Supplementary Files
