# Supplementary material for: An extract from the Atlantic brown algae Saccorhiza polyschides counteracts diet-induced obesity in mice via a gut related multi-factorial mechanisms
Source: Oncotarget. 2017 May 23;8(43):73501–15. doi: 10.18632/oncotarget.18113 (PMC5650277; doi:10.18632/oncotarget.18113)
Supplement: Supplementary file 2 [file oncotarget-08-73501-s002.docx]

Supplementary Tables

**Supplementary Table S1: Concentration of plasma biomarkers in high fat diet fed mice supplemented with a brown algae extract (BAE).** Data are means *± SEM* (n=7). Statistically significant changes (p<0.05) are indicated as *.

|  | Control | BAE |
| --- | --- | --- |
| Total cholesterol [mg/dl] | 171 *± 19.5* | 134 *± 13.6* |
| Triglycerides [mg/dl] | 137 *± 15.0* | 123 *± 22.9* |
| Adiponectin [µg/ml] | 6.7 *± 3.0* | 9.0 *± 3.0* * |
| AST [nmol/min/µl] | 6.2 *± 0.5* | 5.2 *± 0.4* |
| ALT [nmol/min/µl] | 1.8 *± 0.7* | 1.4 *± 0.3* |

**Supplementary Table S2:** **Genes involved in energy homeostasis in brown adipose tissue and skeletal muscle are not modulated in mice fed a high fat diet supplemented with brown algae extract (BAE).** Relative mRNA levels were determined by relating the expression of target to housekeeping genes. Data are means *± SEM* (n=7-8), expressed in relation to the mean of control mice.

| Gene | Name | mRNA level (BAE) |
| --- | --- | --- |
| **Brown adipose tissue** | |  |
| *Ucp1* | uncoupling protein 1 | 1.11 *± 0.18* |
| *Ucp2* | uncoupling protein 2 | 1.32 *± 0.14* |
| *Ucp3* | uncoupling protein 3 | 1.28 *± 0.19* |
| *Dio2* | deiodinase, iodothyronine, type II | 1.19 *± 0.38* |
| *Pparg* | peroxisome proliferator activated receptor gamma, PPARγ | 1.04 *± 0.11* |
| *Ppargc1a* | PPARγ coactivator 1 alpha, PGC1α | 0.93 *± 0.12* |
| **Skeletal muscle** | |  |
| *Ppargc1a* | PPARγ coactivator 1 alpha, PGC1α | 0.87 *± 0.12* |
| *Slc2a4* | solute carrier family 2 member 4 (GLUT4) | 1.05 *± 0.17* |
| *Sirt1* | sirtuin 1 | 0.76 *± 0.09* |

**Supplementary Table S3: LC-quadrupole time of flight mass spectrometer analysis of the secondary metabolites present in brown algae extract, BAE.** Compounds are listed by intensity [counts].

| **Name** | **Sum formula** | **Intensity [counts]** |
| --- | --- | --- |
| Dieckol | C36H22018 | 876 |
| Eckol | C18H12O9 | 513 |
| Ergosterol | C28H44O | 426 |
| 6,6'-Bieckol | C36H22O18 | 413 |
| Diplorethol | C12H10O6 | 406.5 |
| Phloroglucinol | C6H6O3 | 399 |
| Gallic acid | C7H6O5 | 395 |
| 2-O-(2,4,6-trihydroxyphenyl)-6,6'-bieckol | C42H26O21 | 380 |
| Pentfuhalol | C30H22O16 | 365 |
| Fucoxanthin | C42H58O6 | 350 |
| Pentaphlorethol B | C30H22O15 | 345 |
| Hexaphlorethol | C36H26O18 | 288 |
| Bifuhalol trimer | C36H26O21 | 284 |
| 2-Phloroeckol | C24H16O12 | 280 |
| Fucotriphloroethol | C24H18O12 | 273 |
| Bifuhalol | C12H10O7 | 234.5 |
| Bifuhalol tetremer | C48H34O42 | 228 |
| Heptafucol | C42H30O22 | 211 |
| Trifucol | C18H14O9 | 129 |
| Dioxinodehydroeckol | C18H10O9 | 128 |
| Fucosterol | C29H48O | 116 |
| Total intensity |  | 7040 |

**Supplementary Table S4: Sequences of primers used in the SYBR Green RT-PCR analysis.**

| **Gene** | **Forward** | **Reverse** |
| --- | --- | --- |
| Target genes | | |
| *Abcc2* | TCTTCGTCTCCTATGGTTTCCA | CGTGTGTTGAGTCGCTTGATT |
| *Abcc3* | GTCCCCTGCATCTACCTGTG | GCCGTCTTGAGCCTGGATAA |
| *Abcc4* | GCAAATGTGGACCCAAGAAC | TGGTGTTCAGTCTGTGAGCA |
| *Abcg8* | CTGTGGAATGGGACTGTACTTC | TGTTGTCACTTTCCGAGGAGA |
| *Asbt* | CCCAAATGCAACTGTCTGCG | CACCCCATAGAAAACATCACCA |
| *Bsep* | TTCCAGAGGCAGCTATCAGG | CCACAAGCACATCATTGTCC |
| *Cd36* | CAAAACGACTGCAGGTCAAC | CCAATGGTCCCAGTCTCATT |
| *Cyp7a1* | TACAGAGTGCTGGCCAAGAG | GCTGTCCGGATATTCAAGGA |
| *Cyp7b1* | AGGAAAGGCAAGATCTGCTG | GCCCAGAACATAGCTGGAAT |
| *Cyp8b1* | CTAGGGCCTAAAGGTTCGAGT | GTAGCCGAATAAGCTCAGGAAG |
| *Dio2* | acagcttcctcctagatgccta | agtcaagaaggtggcattcg |
| *Dpp4* | CAACCTAACGTGGAACTGTCC | AGCTGCTTCCATCAGAGGTG |
| *Fabp6* | AAGGCTACCGTGAAGATGGA | TCTTGCTTACGCGCTCATAG |
| *Fxr* | CTCCCATTTACAGGCTACGG | GAACTTGAGGAAACGGGACA |
| *G6pc* | tcggagactggttcaacctc | tcacaggtgacagggaactg |
| *Ntcp1* | CTTGCGCCATAGGGATCTT | GTTGCCCACATTGATGACAG |
| *Pck1* | GTATCATCTTTGGTGGCCGTA | ATCTTGCCCTTGTGTTCTGC |
| *Pparg* | AAGAGCTGACCCAATGGTTG | ACCCTTGCATCCTTCACAAG |
| *Ppargc1a* | TGCCCAGATCTTCCTGAACT | TCTGTGAGAACCGCTAGCAA |
| *Scarb1* | GCAAATTTGGCCTGTTTGTT | GATCTTGCTGAGTCCGTTCC |
| *Slc2a4* | GGACCGGATTCCATCCCAC | TCCCAACCATTGAGAAATGATGC |
| *Sirt1* | CAGGTTGCAGGAATCCAAAG | CTCCACGAACAGCTTCACAA |
| *Ucp1* | GAAAGGGACGACCCCTAATC | GGGACGTCATCTGCCAGTA |
| *Ucp2* | GTGGTGGTCGGAGATACCAGA | GGGCAACATTGGGAGAAGTCC |
| *Ucp3* | CTGCACCGCCAGATGAGTTT | ATCATGGCTTGAAATCGGACC |
| Housekeeper genes | | |
| *Act* | GACAGGATGCAGAAGAGATTACT | TGATCCACATCTGCTGGAAGG T |
| *18S* | GGTAACCCGTTGAACCCCAT | CAACGCAAGCTTATGACCCG |
